# The Association Between Parity and Subsequent Cardiovascular Disease in Women: The Atherosclerosis Risk in Communities Study

**DOI:** 10.1089/jwh.2018.7161

**Published:** 2019-05-17

**Authors:** Clare Oliver-Williams, Catherine J. Vladutiu, Laura R. Loehr, Wayne D. Rosamond, Alison M. Stuebe

**Affiliations:** ^1^ Cardiovascular Epidemiology Unit, Department of Public Health and Primary Care, University of Cambridge, Cambridge, United Kingdom.; ^2^ Homerton College, Hills Road, University of Cambridge, Cambridge, United Kingdom.; ^3^ Department of Obstetrics & Gynecology, School of Medicine, University of North Carolina, Chapel Hill, North Carolina.; ^4^ Department of Epidemiology, Gillings School of Global Public Health, University of North Carolina, Chapel Hill, North Carolina.; ^5^ Department of Maternal and Child Health, Gillings School of Global Public Health, University of North Carolina, Chapel Hill, North Carolina.

**Keywords:** pregnancy, breastfeeding, cardiovascular disease

## Abstract

***Background:*** Previous studies are inconclusive on the relationship between parity and cardiovascular disease (CVD), with few evaluating multiple cardiovascular outcomes. It is also unclear if any relationship between parity and CVD is independent of breastfeeding. We examined the associations between parity and cardiovascular outcomes, including breastfeeding adjustment.

***Materials and Methods:*** Data were from 8,583 White and African American women, 45–64 years of age, in the Atherosclerosis Risk in Communities Study. Coronary heart disease (CHD), myocardial infarction (MI), heart failure, and strokes were ascertained from 1987 to 2016 by annual interviews and hospital surveillance. Parity and breastfeeding were self-reported. Cox proportional hazards regression estimated hazard ratios (HR) for the association between parity and cardiovascular outcomes, adjusting for baseline sociodemographic, clinical and lifestyle factors, and breastfeeding.

***Results:*** Women reported no pregnancies (6.0%), or having 0 (1.6%), 1–2 (36.2%), 3–4 (36.4%), or 5+ (19.7%) live births. During 30 years follow-up, there were 1,352 CHDs, 843 MIs, 750 strokes, and 1,618 heart failure events. Compared with women with 1–2 prior births, those with prior pregnancies and no live births had greater incident CHD (HR = 1.64, 95% confidence interval 1.14–2.42) and heart failure risk (1.46, 1.04–2.05), after adjustment for baseline characteristics. Women with 5+ births had greater risk of CHD (1.29, 1.10–1.52) and hospitalized MI (1.38, 1.13–1.69), after adjustment for baseline characteristics and breastfeeding.

***Conclusions:*** In a diverse U.S. cohort, a history of 5+ live births is associated with CHD risk, specifically, MI, independent of breastfeeding. Having a prior pregnancy and no live birth is associated with greater CHD and heart failure risk.

## Introduction

Cardiovascular disease (CVD) is the leading cause of death among women in every major developed country and most emerging economies.^1^ In 2016, ∼296,000 U.S. women died from coronary heart disease (CHD) and an additional 83,000 women died from stroke.^1^ One in three female adults in the United States has some form of CVD; 6.6 million have CHD and 3.8 million are stroke survivors.^2,3^

Pregnancy is a stress test for a woman's body. Physiological changes occurring during pregnancy include weight gain and accumulation of abdominal fat,^4^ higher lipid levels,^5^ increased insulin resistance,^6^ and structural cardiac changes, including increased left ventricular mass and end-systolic volume.^7^ Although most changes that occur during pregnancy are temporary, these changes parallel established CVD risk factors, and thus may have long-term implications for cardiovascular health.^8^ Recognizing that the unique cardiovascular demand that occurs during pregnancy can be used to improve CVD risk prediction, the 2011 American Heart Association guidelines incorporated obstetric complications in the assessment of CVD risk in women and made a call for further research into CVD risk in relation to pregnancy.^9^

Previous studies have assessed the relationship between parity and different cardiovascular outcomes, including CVD,^10^ CHD,^11–13^ and stroke.^12–14^ However, the results of these studies have been conflicting; previous studies have found the lowest risk among nulliparous^10^ or multiparous women,^12^ a J-shaped relationship with the nadir of risk for primiparous women,^14^ or no relationship.^11,13^ This may be due to several reasons, including sociocultural differences in the populations studied and the evaluation of different outcomes. Only a few studies have evaluated multiple cardiovascular outcomes within the same population, making it difficult to confirm the relative size and shape of the relationship across multiple endpoints. These studies have also all had limitations, which include an inability to differentiate nulliparous women who chose not to have children from those who became pregnant, but did not give birth, as a result of a lack of detailed information^12–14^ or a small sample size.^10,11^ Furthermore, few studies have adjusted for breastfeeding, in spite of a possible cardioprotective effect,^15^ which may be due to decreased risk of type 2 diabetes, hyperlipidemia, and hypertension in women who breastfeed.^15^

To address the limitations of previous studies and inform preventive efforts to reduce CVD incidence among women, we sought to examine the association between parity and several clinically relevant CVD outcomes among a cohort of women enrolled in the Atherosclerosis Risk in Communities (ARIC) Study, while also adjusting for breastfeeding. We hypothesized that women with no prior live births and those with three or more prior live births would have a higher incidence of overall CVD and its clinical subtypes compared with those with one to two prior live births, with a J-shaped association expected overall.

## Materials and Methods

### Study population

The ARIC study is a multicenter, population-based, prospective cohort study of middle-aged adults from four U.S. communities: Forsyth County NC, Jackson MS, suburban Minneapolis MN, and Washington County MD. Briefly, 15,792 men and women, 45–64 years of age, were recruited to participate and had their first clinic examination during 1987–1989. Follow-up examinations were conducted in 1990 to 1992 (visit 2), 1993 to 1995 (visit 3), 1996 to 1998 (visit 4), 2011 to 2013 (visit 5), and 2016 to 2017 (visit 6). In addition, there were annual follow-up interviews to ascertain vital status and document medical and life course events. Participants were predominantly White or African American. Details about the study design and methods have been described previously.^16^ For this analysis, we excluded men (*n* = 7,082), women who did not provide information on live births (*n* = 74), and those without any follow-up (*n* = 1). To reduce potential residual confounding from racial and socioeconomic differences in exposure and outcome, women who self-reported their race as other than White or African American (*n* = 26), and the small number of African Americans in the Minnesota and Washington County cohorts were also excluded (*n* = 10 and *n* = 17, respectively).

This resulted in a final cohort of 8,583 women. Institutional Review Boards at each clinical site approved the study protocol, and written informed consent was obtained from all participants.

### Measures

#### Parity

At visit 1, women were asked to report the total number of live births (parity) in an interviewer-administered questionnaire. Subsequently at visit 3, women were asked whether they had ever been pregnant (gravidity), and the total number of months that they breastfed. We used parity and gravidity to categorize women into five mutually exclusive groups: never pregnant, pregnant with no live births, 1–2 births, 3–4 births, and 5+ births.

#### Covariates

Women reported their age, race, and health insurance status during an interview-administered questionnaire at visit 1. They also reported cigarette smoking status (current, former, or never), and reproductive history (use of birth control pills, use of hormone replacement therapy [HRT], age at menarche, and menopausal status). Socioeconomic status was calculated from the area deprivation index using the 2000 Census, which represents a geographic area-based measure of the socioeconomic deprivation experienced by a neighborhood based on a range of factors, including employment, regional income, and vehicle ownership. Higher scores indicate greater deprivation.^17^ Neighborhood deprivation was included instead of individual markers to ensure adjustment for social and cultural factors known to be associated with both pregnancy^18^ and cardiovascular outcomes,^19^ while also indirectly measuring some individual markers, for example, education.

Years of reproductive life were calculated as the years from menarche to either menopause, or age at baseline for premenopausal women. Age at first pregnancy was abstracted from the visit 3 interview-administered questionnaire. Decade of menarche was calculated from self-reported age at menarche, as reported at visit 1, and date of birth. Average duration of breastfeeding per child, henceforth referred to as breastfeeding duration, was calculated per child by dividing the total duration of breastfeeding, reported in months, by the number of prior live births and was categorized as: no breastfeeding, <3 months, 3–6 months, and 6+ months.

A combined race-center variable was generated because of the disparate distribution of race groups across the ARIC centers. The race-center variable categorized the White and African American participants separately for each center: African American Forsyth County, African American Jackson, White Forsyth County, White Minneapolis, and White Washington County.

At baseline (visit 1), weight and height were measured by trained technicians at the clinical exam. Weight at age 25 was also self-reported at baseline. These were used to calculate BMI at baseline and BMI at age 25 with the following equation: weight(kg)/height^2^(m^2^). Plasma cholesterol and triglycerides were measured from blood samples collected at visit 1, as previously described.^16^

#### Cardiovascular events

The outcomes of interest were cardiovascular events, the ascertainment of which has been detailed previously.^20,21^ Participants reported cardiovascular hospitalizations annually, which were verified and augmented by community-wide hospital surveillance of death certificates and physician review of hospital records. CHD was defined as a definite or probable myocardial infarction (MI), fatal CHD, or silent MI, as ascertained from electrocardiographic evidence. Definite or probable stroke, both hemorrhagic and ischemic, hospitalized MI, and heart failure were also examined. A computer algorithm and physician reviewer classified events as definite or probable. A composite outcome of both CHD and stroke was derived from CHD and definite or probable stroke, and thus combined fatal CHD, MI, ischemic, and hemorrhagic stroke. Follow-up data for cardiovascular events were available through December 31, 2016.

### Missing data

Missing data were imputed using multiple imputation by chained equations for socioeconomic deprivation, health insurance, smoking status, years of reproductive life, oral contraceptive use, HRT use, decade of menarche, age at first pregnancy, duration of breastfeeding, and weight at age 25.^22^ Five imputations were created using a set of appropriate imputation models constructed of all covariates and outcome variables.

### Statistical analyses

Baseline characteristics of the study population were compared by parity categories. Continuous variables were summarized by median and interquartile interval, whereas categorical variables were summarized by number and percentage.

Cox proportional hazards regression models estimated the risk of cardiovascular events with time since baseline as the underlying time scale. The proportional hazards assumption was assessed using the test of Grambsch and Therneau in nonimputed data.^23^ Hazard ratios (HR) and 95% confidence intervals (CI) were calculated for the risk of incident cardiovascular outcomes by parity categories, with one to two births as the reference category chosen to assess the hypothesis that nulliparous and multiparous women have a higher incidence of cardiovascular outcomes.

The association between parity and the risk of five outcomes, composite CHD and stroke; CHD; hospitalized MI; heart failure, and stroke, was estimated in four models progressively adjusting for covariates. These were: model 1 (age), model 2 (age, race-center, socioeconomic deprivation, health insurance, and smoking status), model 3 (covariates from model 2 plus duration of reproductive life, oral contraceptive pill and HRT use, and decade of menarche), and model 4 (covariates from model 3 plus age at first pregnancy and breastfeeding duration). Women with no prior pregnancies were not included in model 4 since they did not have an age at first pregnancy or breastfeeding duration.

Several sensitivity analyses were conducted. To assess for any racial differences in the associations, results were stratified by race. Analyses were also conducted, including the covariates from model 3, plus BMI at age 25 to assess the robustness of the findings to confounding by BMI closer to the time of pregnancy. As it is unclear if hyperlipidemia, diabetes, and hypertension are intermediates on the causal pathway or confounders, two additional sensitivity analyses were conducted. Women with diabetes or hypertension at baseline were excluded (*n* = 998 and 2,823, respectively), and thus any women who had diabetes or hypertension before pregnancy. In separate analyses, we also adjusted for baseline total cholesterol and triglycerides.

*p*-Values for all hypothesis tests were two-sided, and statistical significance was set at *p* < 0.05. All statistical analyses were performed using the Stata software package, version 14.2 (Stata Corporation, College Station, TX).

## Results

Women reported never being pregnant (6.0%), or having been pregnant and having had none (1.6%), 1–2 (36.2%), 3–4 (36.4%), or 5+ (19.7%) live births (Table 1). A high percentage of women with prior pregnancies and no live births, and women with 5+ births were more likely to be African American, to be uninsured, and to have a higher BMI. They also had higher socioeconomic deprivation as indicated by higher scores. Women with 5+ births were younger at first pregnancy (mean: 19 years), and women with no live births were less likely to have used oral contraceptives.

**Table 1.** Maternal Characteristics by Parity Categories: The ARIC Cohort Study, 1987–2016

	*Never pregnant*	*No. of live births in women with prior pregnancies*			
		*0*	*1–2*	*3–4*	*5+*
*N* (%)	517 (6.0)	138 (1.6)	3,108 (36.2)	3,126 (36.4)	1,694 (19.7)
Age, years	54 (49–60)	55 (49–60)	53 (48–58)	53 (49–58)	55 (51–59)
Socioeconomic deprivation^a,b^	104.1 (97.4–109.2)	110.1 (102.5–118.5)	104.3 (98.1–109.2)	104.0 (97.9–110.8)	112.9 (103.1–118.5)
Race and center, *n* (%)					
African American, Forsyth County	17 (3.3)	12 (8.7)	84 (2.7)	89 (2.9)	81 (4.8)
African American, Jackson	91 (17.6)	61 (44.2)	605 (19.5)	685 (21.9)	853 (50.4)
White, Forsyth County	151 (29.2)	23 (16.7)	916 (29.5)	636 (20.4)	140 (8.3)
White, Minneapolis	119 (23.0)	21 (15.2)	658 (21.2)	916 (29.3)	326 (19.2)
White, Washington County	139 (26.9)	21 (15.2)	845 (27.2)	800 (25.6)	294 (17.4)
Health insurance, *n* (%)	482 (93.2)	120 (87.0)	2,886 (93.0)	2,853 (91.4)	1,318 (77.9)
Current smoker, *n* (%)	126 (24.4)	39 (28.5)	829 (26.7)	726 (23.3)	404 (23.9)
Oral contraceptives (ever)^a^, *n* (%)	145 (28.1)	41 (29.3)	1,417 (45.6)	1,556 (49.8)	702 (41.4)
HRT^a^, *n* (%)					
Estrogen	74 (14.8)	17 (13.0)	428 (14.2)	417 (13.8)	178 (11.0)
Estrogen and progestin	35 (7.0)	8 (6.1)	182 (6.0)	189 (6.3)	39 (2.4)
Never user	320 (64.2)	84 (64.1)	1,983 (65.9)	2,024 (67.0)	1,186 (73.1)
Former user	70 (14.0)	22 (16.8)	418 (13.9)	391 (12.9)	219 (13.5)
Years of reproductive life	33 (29–37)	32 (28–36)	33 (29–36)	34 (29–37)	34 (29–37)
Age at first pregnancy, years	—	24 (20–30)	22 (20–26)	21 (19–23)	19 (17–21)
Decade of menarche^a^, *n* (%)					
1930	88 (17.2)	24 (17.7)	431 (13.9)	374 (12.0)	256 (15.1)
1940	248 (48.6)	69 (50.7)	1,400 (45.2)	1,555 (49.8)	954 (56.4)
1950	172 (33.7)	43 (31.6)	1,263 (40.8)	1,185 (38.0)	481 (28.4)
1960	2 (0.4)	0 (0.0)	5 (0.2)	8 (0.3)	2 (0.1)
Breastfeeding duration, *n* (%)					
0 months	517 (100.0)	138 (100.0)	1,836 (63.6)	1,472 (50.8)	531 (35.7)
<3 months	0 (0.0)	0 (0.0)	461 (16.0)	798 (27.6)	497 (33.4)
3–6 months	0 (0.0)	0 (0.0)	308 (10.7)	342 (11.8)	215 (14.5)
6+ months	0 (0.0)	0 (0.0)	283 (9.8)	285 (9.8)	244 (16.4)
BMI at age 25, kg/m^2^	20.9 (19.5–22.5)	21.2 (19.5–23.3)	21.0 (19.6–22.8)	21.3 (19.8–23.1)	21.7 (20.0–23.8)
BMI at baseline, kg/m^2^	25.7 (22.4–29.6)	28.1 (23.7–33.2)	25.6 (22.6–29.7)	26.7 (23.6–31.3)	29.3 (25.3–34.0)
HDL cholesterol, mmol/L	1.54 (1.22–1.84)	1.38 (1.15–1.84)	1.44 (1.20–1.77)	1.42 (1.15–1.73)	1.37 (1.15–1.67)
LDL cholesterol, mmol/L	3.47 (2.80–4.20)	3.42 (2.89–3.97)	3.39 (2.78–4.07)	3.41 (2.80–4.15)	3.53 (2.88–4.24)
Triglycerides, mmol/L	1.05 (0.80–1.55)	1.25 (0.81–1.81)	1.15 (0.85–1.64)	1.19 (0.85–1.67)	1.21 (0.89–1.69)
Diabetes at baseline, *n* (%)	46 (9.0)	34 (25.0)	285 (9.2)	324 (10.5)	309 (19.6)
Hypertension at baseline, *n* (%)	152 (29.5)	78 (56.5)	963 (31.1)	1,007 (32.4)	815 (48.3)
CHD and stroke, *n* (%)	106 (20.5)	40 (29.0)	605 (19.5)	661 (21.5)	478 (28.2)
CHD, *n* (%)	77 (14.9)	30 (21.7)	435 (14.0)	472 (15.1)	338 (20.0)
Hospitalized myocardial infarction, *n* (%)	47 (9.1)	15 (10.9)	269 (8.7)	278 (8.9)	234 (13.8)
Stroke, *n* (%)	37 (7.2)	15 (10.9)	229 (7.4)	263 (8.4)	206 (12.2)
Heart failure^a^, *n* (%)	98 (20.1)	37 (29.8)	531 (18.6)	535 (18.6)	417 (27.2)

Data presented are median (interquartile interval) unless otherwise stated. Prevalent heart failure cases are coded as missing.

^a^ Missing data: socioeconomic deprivation—172 (2.00%), health insurance—12 (0.14%), smoking—12 (0.14%), years of reproductive life—498 (3.78%), age at first pregnancy—591 (6.86%), oral contraceptives (ever)—4 (0.05%), HRT use—299 (3.47%), decade of menarche—23 (0.27%), breastfeeding duration—663 (7.70%), BMI at age 25—115 (1.34%), BMI at baseline—7 (0.1%), HDL cholesterol—160 (1.9%), LDL cholesterol—252 (2.9%), triglycerides—160 (1.9%), and heart failure—634 (7.36%).

^b^ Higher scores indicate greater deprivation.

BMI, body mass index; CHD, coronary heart disease; HDL, high-density lipoprotein; HRT, hormone replacement therapy; LDL, low-density lipoprotein.

Women with prior pregnancies and no live births and those with 5+ births had a greater risk of composite CHD and stroke compared with women with one to two births, after adjustment for age (HR [model 1] = 1.65, 95% CI 1.20–2.28 and HR [model 1] = 1.44, 95% CI 1.26–1.63, respectively) (Table 2). Adjustment for sociodemographic, behavioral, and reproductive characteristics reduced the magnitude of the associations, HR (model 3) = 1.49, 95% CI 1.08–2.05 and HR (model 3) = 1.32; 95% CI 1.16–1.50, respectively. Minimal attenuation for women with 5+ births was observed after further adjustment for breastfeeding and age at first pregnancy, HR (model 4) = 1.26, 95% CI 1.10–1.44.

**Table 2.** Hazard Ratios and 95% Confidence Intervals for the Association Between Parity Categories and Cardiovascular Outcomes: The ARIC Cohort Study, 1987–2016

	*Never pregnant* n* = 517*	*No. of live births in women with prior pregnancies*			
		*0*	*1–2*	*3–4*	*5+*
		n* = 138*	n* = 3,108*	n* = 3,126*	n* = 1,694*
Composite CHD and stroke					
Events/person-years	106/11,897	40/2,628	605/69,316	661/69,887	478/35,386
Model 1	0.95 (0.78–1.17)	1.65 (1.20–2.28)	1 (ref.)	1.08 (0.97–1.20)	1.44 (1.26–1.63)
Model 2	0.95 (0.78–1.18)	1.50 (1.09–2.07)	1 (ref.)	1.11 (0.99–1.24)	1.28 (1.13–1.46)
Model 3	0.95 (0.77–1.18)	1.49 (1.08–2.05)	1 (ref.)	1.13 (1.01–1.26)	1.32 (1.16–1.50)
Model 4	—	—	1 (ref.)	1.09 (0.97–1.22)	1.26 (1.10–1.44)
CHD					
Events/person-years	77/12,035	30/2,680	435/70,581	472/70,979	338/36,364
Model 1	0.98 (0.77–1.25)	1.72 (1.18–2.49)	1 (ref.)	1.07 (0.94–1.22)	1.41 (1.22–1.63)
Model 2	0.99 (0.78–1.27)	1.66 (1.14–2.42)	1 (ref.)	1.12 (0.98–1.28)	1.34 (1.16–1.57)
Model 3	0.99 (0.77–1.26)	1.64 (1.14–2.39)	1 (ref.)	1.14 (1.00–1.30)	1.39 (1.19–1.61)
Model 4	—	—	1 (ref.)	1.09 (0.95–1.25)	1.29 (1.10–1.52)
Hospitalized myocardial infarction					
Events/person-years	47/12,308	15/2,828	269/72,103	278/72,555	234/37,137
Model 1	0.96 (0.71–1.31)	1.35 (0.89–2.26)	1 (ref.)	1.35 (0.80–2.26)	1.58 (1.32–1.88)
Model 2	0.98 (0.72–1.34)	1.25 (0.74–2.11)	1 (ref.)	1.07 (0.90–1.27)	1.46 (1.22–1.76)
Model 3	0.97 (0.71–1.32)	1.23 (0.73–2.08)	1 (ref.)	1.09 (0.92–1.29)	1.50 (1.24–1.80)
Model 4	—	—	1 (ref.)	1.04 (0.87–1.24)	1.38 (1.13–1.69)
Heart failure					
Events/person-years	98/11,596	37/2,490	531/67,714	535/67,293	417/33,443
Model 1	0.99 (0.80–1.23)	1.81 (1.29–2.52)	1 (ref.)	1.00 (0.89–1.13)	1.48 (1.30–1.68)
Model 2	1.03 (0.83–1.27)	1.49 (1.06–2.09)	1 (ref.)	1.04 (0.92–1.17)	1.22 (1.06–1.17)
Model 3	1.01 (0.81–1.25)	1.46 (1.04–2.05)	1 (ref.)	1.06 (0.94–1.20)	1.25 (1.09–1.43)
Model 4	—	—	1 (ref.)	1.03 (0.90–1.16)	1.17 (1.01–1.36)
Stroke					
Events/person-years	37/12,503	15/2,871	229/72,509	263/73,382	206/37,329
Model 1	0.87 (0.61–1.23)	1.56 (0.92–2.63)	1 (ref.)	1.12 (0.94–1.34)	1.62 (1.34–1.96)
Model 2	0.87 (0.62–2.05)	1.21 (0.72–2.05)	1 (ref.)	1.12 (0.94–1.34)	1.22 (1.00–1.50)
Model 3	0.85 (0.60–1.21)	1.19 (0.70–2.02)	1 (ref.)	1.15 (0.96–1.37)	1.27 (1.04–1.55)
Model 4	—	—	1 (ref.)	1.13 (0.94–1.36)	1.25 (1.00–1.55)

Model 1—age.

Model 2—Model 1+combined race and center, socioeconomic deprivation, health insurance, smoking status.

Model 3—Model 2+years of reproductive life, use of the oral contraceptive pill (ever), HRT use, decade of menarche.

Model 4—Model 3+age at first pregnancy and breastfeeding duration per child.

When assessing individual cardiovascular events, women with prior pregnancies and no live births and those with 5+ births had an increased risk of at least one outcome, compared with women with 1–2 births (Table 2). Women with prior pregnancies and no live births were at greater risk of both CHD, HR (model 3) = 1.64, 95% CI 1.14–2.39, and heart failure, HR (model 3) = 1.46, 95% CI 1.04–2.05, after adjustment for age, sociodemographic, behavioral, and reproductive factors. Women with 5+ births had a higher risk of CHD, HR (model 4) = 1.29, 95% CI 1.10–1.52, and hospitalized MI, HR (model 4) = 1.38, 95% CI 1.13–1.69, after adjustment for all factors, including age at first pregnancy and breastfeeding duration.

Sensitivity analyses adjusting for BMI at age 25 or baseline total cholesterol and triglycerides, in addition to the sociodemographic, behavioral, and reproductive characteristics in Model 3, had negligible impact on the results. As did exclusion of women with diabetes at baseline (*n* = 998, 12% of cohort) or hypertension at baseline (*n* = 2,823, 33% of cohort). Stratifying analyses by race showed no discernible differences in risk (results available upon request).

## Discussion

In this analysis of a diverse cohort of women in four U.S. communities, we estimated the relationship between parity and several cardiovascular outcomes. Women with prior pregnancies and no live births were at greater risk of both CHD and heart failure. Women with 5+ births had a higher risk of incident CHD and hospitalization for MI, independent of breastfeeding.

The increased cardiovascular risk associated with multiple births has been found previously,^10,14^ although neither study adjusted for duration of breastfeeding per child. There are several possible mechanisms that may explain this association. There is evidence that repeated pregnancies could result in cardiometabolic changes, including weight gain,^24^ increased waist circumference,^24^ and hyperlipidemia,^5^ as well as subclinical atherosclerosis.^25^ These previous findings are reflected in our own cohort, where women with multiple births had a higher BMI, lower high-density lipoprotein cholesterol, higher triglycerides, and higher low-density lipoprotein cholesterol at baseline (visit 1) than women with fewer births (Table 1). The observed association may also derive from pregnancy-related changes, including increased left ventricular mass and end-systolic volume,^7^ although an association between increasing number of offspring and greater CVD risk has also been found in men.^26^ This latter finding raises the possibility of socioeconomic factors as a mechanism. Indeed, lower socioeconomic status is associated with a larger family size^27^ as well as greater CVD risk factors.

However, as parity is a proxy for multiple factors (including socioeconomic deprivation, age at menopause, and health conditions) and it comprises both pregnancy and child rearing, it is unclear from our analysis whether these changes in CVD risk factors reflect the direct impact of physiological changes from multiple pregnancies, or whether the changes reflect stressors associated with rearing multiple children. The negligible impact of adjustment for breastfeeding duration, however, suggests that the differences in risk between the groups are not due to breastfeeding. An additional analysis using the variables in model 3 while also adjusting for BMI at age 25 had no material impact on the results, thus indicating that BMI close to the time of pregnancy did not confound the association.

The increased risk of CHD and heart failure found in women with prior pregnancies, but no previous births may reflect the increased risk of CHD that has been previously identified after a history of miscarriage.^28^ Many of the women with prior pregnancies but no births will have experienced miscarriages (as opposed to stillbirths, which occur less frequently^29,30^). Thus, the mechanisms proposed to underlie the relationship between miscarriage and CHD, including immune disorders, endothelial dysfunction, and chronic diseases,^28^ may also underpin the increased risk identified in the current analysis. Of note, women who only underwent therapeutic abortions would also be categorized as having prior pregnancies but no previous births.

Our results are in contrast to some previous studies that found either an increase CVD risk for all nulliparous women,^12,31^ or no increased risk of particular cardiovascular outcomes, such as heart failure^32^ and CHD.^33^ This may be explained by distinguishing women with prior pregnancies, but no live births, from those who had never been pregnant. If women with prior pregnancies are at increased cardiovascular risk, but women who have never been pregnant are not, then combining these two groups may either give the impression that nulliparous women, as a whole, are at greater cardiovascular risk, or may diminish any increased risk.

There is a clear need for accurate assessment of CVD risk in women as it is the leading cause of death; one in three U.S. women die from CVD each year.^2^ Parity is a clinically useful measurement that may be beneficial in CVD risk prediction. However, to ensure this finding is of public health importance, the results need to be replicated in other large, diverse datasets of women with detailed parity information.

The strengths and potential limitations of our study merit consideration. This analysis included a large sample of middle-aged women, with a long duration of follow-up (mean: 23.7 years, maximum: 30.1 years). This study measured anthropometrics and collected detailed information on reproductive history, allowing for further assessment of the potential confounding or mediating role of these relevant factors and, unlike the majority of previous studies on parity, did collect information on breastfeeding.

Importantly, the heterogeneous nulliparous group was separated into those individuals who had been pregnant but did not have a live birth and those who have never been pregnant, allowing assessment of the potential impact of this differentiation on CVD risk.

This study has limitations. There was no information on pregnancy outcomes and complications, such as preterm birth, gestational hypertension, or pre-eclampsia, all of which have been associated with an increased CVD risk.^34–36^ It is not clear if BMI, hyperlipidemia, diabetes, or hypertension status are confounders or intermediates on the causal pathway. However, adjustment for neither BMI at age 25, nor baseline total cholesterol and triglycerides, affected the findings. As the timing of hypertension and diabetes diagnoses with respect to pregnancy was unknown, women with diabetes or hypertension at baseline were excluded to assess the relationship in an otherwise health population. This also had negligible impact on the results. Finally, there was also a risk of residual confounding by socioeconomic deprivation. Marked differences between parity categories were noted for race and center in combination and insurance status, suggesting a cumulative exposure to adverse social determinants of health, which may not have been entirely captured by the socioeconomic measures included in the current analysis.

Furthermore, the analysis was limited to White and African American participants, thus the results may not be generalizable to other ethnicities. It was not possible to differentiate between therapeutic abortions, miscarriages, and stillbirths for women who reported prior pregnancies and no live births, making it unclear whether all nulliparous women with previous pregnancies are at greater risk of CVD. Breastfeeding was self-reported and may be misclassified due to recall bias. Secular trends during the lifetime of study participants may also have affected results, as hormonal birth control became widely available in 1960, altering childbearing patterns in the United States.

## Conclusions

The main finding of our study is that women with prior pregnancies, but no live births, are at greater risk of incident CHD and heart failure, whereas women with five or more live births are at greater risk of CHD and hospitalization from MI, independent of breastfeeding. Whether this increased risk is due to the direct impact of pregnancy or through lifestyle behavior changes is unclear, and requires further research.
